# The Novel Oxazolidinone TBI-223 Is Effective in Three Preclinical Mouse Models of Methicillin-Resistant Staphylococcus aureus Infection

**DOI:** 10.1128/spectrum.02451-21

**Published:** 2022-09-15

**Authors:** Oren Gordon, Dustin A. Dikeman, Roger V. Ortines, Yu Wang, Christine Youn, Mohammed Mumtaz, Nicholas Orlando, Jeffrey Zhang, Aman M. Patel, Ethan Gough, Amit Kaushik, Eric L. Nuermberger, Anna M. Upton, Nader Fotouhi, Lloyd S. Miller, Nathan K. Archer

**Affiliations:** a Division of Infectious Diseases, Department of Pediatrics, Johns Hopkins University School of Medicine, Baltimore, Maryland, USA; b Department of Dermatology, Johns Hopkins University School of Medicine, Baltimore, Maryland, USA; c Department of International Health, Johns Hopkins Bloomberg School of Public Health, Baltimore, Maryland, USA; d Center for Tuberculosis Research, Johns Hopkins University grid.471401.7grid.21107.35grid.471401.7 , Baltimore, Maryland, USA; e Evotec (US) Inc., Princeton, New Jersey, USA; f TB Alliance, New York, New York, USA; g Immunology, Janssen Research and Development, Spring House, Pennsylvania, USA; Emory University School of Medicine

**Keywords:** *Staphylococcus aureus*, antibiotic resistance, oxazolidinones

## Abstract

Staphylococcus aureus is an important cause of various infections in humans, including bacteremia, skin and soft tissue infections, and infections associated with implanted medical devices. The emergence of hospital- and community-acquired methicillin-resistant Staphylococcus aureus (MRSA) underscores the urgent and unmet need to develop novel, safe, and effective antibiotics against these multidrug-resistant clinical isolates. Oxazolidinone antibiotics such as linezolid have excellent oral bioavailability and provide coverage against MRSA infections. However, their widespread and long-term use is often limited by adverse effects, especially myelosuppression. TBI-223 is a novel oxazolidinone with potentially reduced myelosuppression, compared to linezolid, but its efficacy against MRSA infections is unknown. Therefore, the preclinical efficacy of TBI-223 (80 and 160 mg/kg twice daily) was compared with that of linezolid (40 and 80 mg/kg twice daily) and sham treatment in mouse models of MRSA bacteremia, skin wound infection, and orthopedic-implant-associated infection. The dosage was selected based on mouse pharmacokinetic analysis of both linezolid and TBI-223, as well as measurement of the MICs. In all three models, TBI-223 and linezolid had comparable dose-dependent efficacies in reducing bacterial burden and disease severity, compared with sham-treated control mice. Taken together, these findings indicate that TBI-223 represents a novel oxazolidinone antibiotic that may provide an additional option against MRSA infections. Future studies in larger animal models and clinical trials are warranted to translate these findings to humans.

**IMPORTANCE** Staphylococcus aureus is the predominant cause of bloodstream, skin, and bone infections in humans. Resistance to commonly used antibiotics is a growing concern, making it more difficult to treat staphylococcal infections. Use of the oxazolidinone antibiotic linezolid against resistant strains is hindered by high rates of adverse reactions during prolonged therapy. Here, a new oxazolidinone named TBI-223 was tested against S. aureus in three mouse models of infection, i.e., bloodstream infection, skin infection, and bone infection. We found that TBI-223 was as effective as linezolid in these three models. Previous data suggest that TBI-223 has a better safety profile than linezolid. Taken together, these findings indicate that this new agent may provide an additional option against MRSA infections. Future studies in larger animal models and clinical trials are warranted to translate these findings to humans.

## INTRODUCTION

Staphylococcus aureus is a Gram-positive bacterial human pathogen that is an important cause of various clinical infections, including bacteremia, skin and soft tissue infections (SSTIs), and orthopedic-implant-associated infections (OIAIs) (1). The widespread emergence of hospital- and community-acquired methicillin-resistant S. aureus (MRSA) strains that are resistant to multiple antibiotics, along with the limited development of new antibiotics in the pipeline, is creating a serious public health threat.

Oxazolidinones are a class of antibiotics with activity against Gram-positive bacteria, including MRSA, that inhibit protein synthesis by the unique binding to a distinct region of 23S RNA adjacent to the peptidyl transferase center of the bacterial 50S ribosomal subunit (2, 3). However, the widespread and prolonged use of oxazolidinone antibiotics has been limited because of adverse events such as myelosuppression, monoamine oxidase inhibition, and neuropathy associated with mitochondrial protein synthesis (MPS). These effects are likely mediated by the homology between the 23S ribosomal bacterial target and the closely related MPS machinery in mammals (4, 5). For example, linezolid is a clinically used oxazolidinone with high oral bioavailability and penetration into target tissues, including skin and bone (6, 7), with significant efficacy against MRSA bacteremia, SSTIs, and OIAIs (6, 8–14). However, linezolid use has increased risk of significant adverse effects during prolonged courses (e.g., >2 weeks), which restricts its usage (7, 15, 16). If the associated adverse side effects of oxazolidinone antibiotics could be mitigated, then this could greatly improve their utility against MRSA infections, especially for infections such as bacteremia and OIAIs, which often require prolonged courses of antibiotic therapy.

TBI-223 is a novel oxazolidinone with excellent oral bioavailability and activity against Gram-positive bacteria (17, 18); it has been shown to have reduced human and mouse bone marrow progenitor cell toxicity *in vitro* and bone marrow toxicity in rat and dog models *in vivo*, compared to linezolid (as measured by bone marrow histopathology and platelet and reticulocyte counts) (19, 20). Moreover, TBI-223 is being evaluated in phase I clinical studies for treatment of tuberculosis (21). However, the *in vivo* efficacy of TBI-223 against MRSA infections is unknown. Therefore, we investigated the *in vivo* efficacy of TBI-223 in comparison with linezolid in three different mouse models of MRSA infection, namely, bacteremia, SSTIs, and OIAIs.

## RESULTS

### Mouse pharmacokinetic analysis, MICs, and human equivalent dosing.

A mouse pharmacokinetic (PK) study was done to determine blood drug concentrations up to 24 h following a single oral dose of 100 mg/kg of either linezolid or TBI-223 (Table 1; also see Tables S1 and S2 in the supplemental material). The area under the concentration-time curve (AUC) for linezolid was 130.7 ± 8.5 μg · h/mL and the half-life (*t*_1/2_) was 1.58 ± 0.4 h, similar to previously published data (22, 23). The AUC for TBI-223 was 179.4 ± 19.1 μg · h/mL and the *t*_1/2_ was 3.0 ± 0.4 h. Assuming dose proportionality, we determined that a twice-daily dose of linezolid (80 mg/kg) in mice would provide an equivalent AUC, compared with the clinically used twice-daily dose of linezolid (600 mg) in humans (AUC values of 209.12 μg · h/mL and 228 μg · h/mL, respectively) (10, 22–24).

**TABLE 1:** PK analysis of linezolid and TBI-223 in mice

Parameter^*a*^	Data (mean ± SD) for:	
	Linezolid	TBI-223
AUC_0–24_ (h · μg/mL)	130.7 ± 8.5	179.4 ± 19.1
*C*_max_ (μg/mL)	52.6 ± 6.4	44.5 ± 7.1
*t*_1/2_ (h)	1.6 ± 0.4	3.0 ± 0.4

a Drug plasma concentrations were measured 0.25, 0.5, 1, 2, 4, 7, and 24 h after an oral dose of 100 mg/kg of either linezolid or TBI-223.

Next, we compared the MICs of TBI-223 and linezolid in linezolid-susceptible (*n* = 5) and linezolid-resistant (*n* = 5) clinical strains using broth microdilution (see Table S3). We found that the MIC for TBI-223 was 4 times higher than the MIC for linezolid in all susceptible strains and all linezolid-resistant strains had high MICs for TBI-223 (>16 μg/mL). We then determined the MICs in a bioluminescent MRSA strain (SAP231) (derived from the community-acquired MRSA parental strain USA300 LAC) (25) and found that the MICs for TBI-223 and linezolid were 4 μg/mL and 1 μg/mL, respectively, consistent with our findings for other linezolid-susceptible strains.

To compensate for the 4-fold difference in MIC values and to evaluate potential equivalent outcomes between linezolid and TBI-223, we used a 2-fold higher dose of TBI-223 (160 mg/kg), compared to linezolid (80 mg/kg), which took into consideration the 2-fold longer half-life of TBI-223 (Table 1). In addition, lower doses of linezolid (40 mg/kg) and TBI-223 (80 mg/kg) were evaluated to determine potential dose-dependent effects.

### Comparison of TBI-223 and linezolid efficacies in a mouse bacteremia infection model.

**FIG 1:**
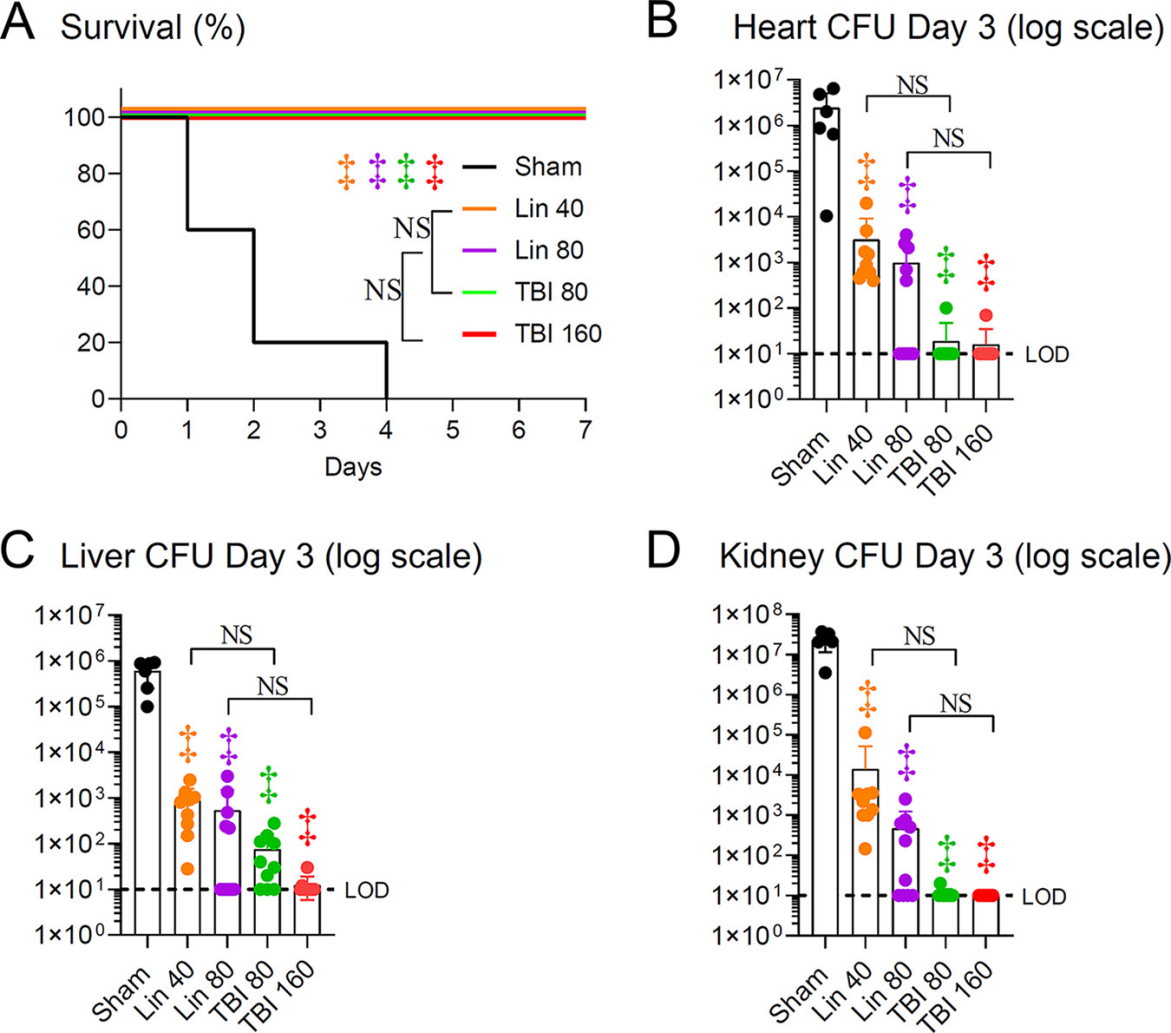
TBI-223 (TBI) versus linezolid (Lin) in a mouse model of MRSA bacteremia. Mice were inoculated IV with 5 × 10^7^ CFUs of SAP231 via the retro-orbital vein. Antibiotic treatment was started 4 h post-infection and continued every 12 h up to 7 days. Treatment groups included linezolid at 40 mg/kg/dose, linezolid at 80 mg/kg/dose, TBI-223 at 80 mg/kg/dose, TBI-223 at 160 mg/kg/dose, and sham treatment (vehicle). (A) Kaplan-Meier graph of percent survival (representative results of one of two independent experiments; *n* = 10 mice/group). (B to D) CFUs (mean ± standard error of the mean [SEM]) from heart (B), liver (C), and bilateral kidneys (D) harvested from euthanized mice that survived to day 3 in a subsequent experiment (combined data from two independent experiments; *n* = 9 or 10 per treatment group, with 6 surviving mice in the sham treatment group). The limit of detection (LOD) was 10 CFUs per organ. ‡, *P < *0.001, antibiotic versus sham treatment groups (symbols) or the indicated groups (brackets) by the log-rank (Mantel-Cox) test (A) or one-way ANOVA (B to D), both adjusted for multiple comparisons to preserve the desired false-discovery rate. NS, nonsignificant.

We compared the efficacy of TBI-223 with that of linezolid in a MRSA bacteremia infection model (26), whereby C57BL/6 mice were injected intravenously (IV) with the community-acquired MRSA strain SAP231 and survival was monitored over time. The mice were treated with linezolid (40 and 80 mg/kg) or TBI-223 (80 and 160 mg/kg) or sham initiated 4 h after inoculation, and treatment was continued twice daily until the experiment was arbitrarily ended on day 7. Sham-treated mice all succumbed to the infection (0% survival) by day 4, whereas linezolid (40 and 80 mg/kg)-treated and TBI-223 (80 and 160 mg/kg)-treated mice all survived (100% survival) through the end of the experiment (Fig. 1A). Next, for mice that survived until day 3, heart, liver, and kidneys were harvested, and there were significantly lower CFU counts in linezolid (40 and 80 mg/kg)-treated and TBI-223 (80 and 160 mg/kg)-treated mice than in sham-treated mice (*P < *0.001) (Fig. 1B to D). However, there were no statistically significant differences in comparisons of 80 versus 160 mg/kg and 40 versus 80 mg/kg linezolid versus TBI-223, respectively. Survival was also evaluated with another MRSA strain (AR-997) (see Table S3) in the bacteremia model. While this strain was less virulent (56% survival rate for sham-treated mice at day 10), both linezolid and TBI-223 treatment fully protected mice and resulted in 100% survival (see Fig. S1).

### Efficacies of TBI-223 and linezolid in a nondiabetic mouse model of skin wound infection.

**FIG 2:**
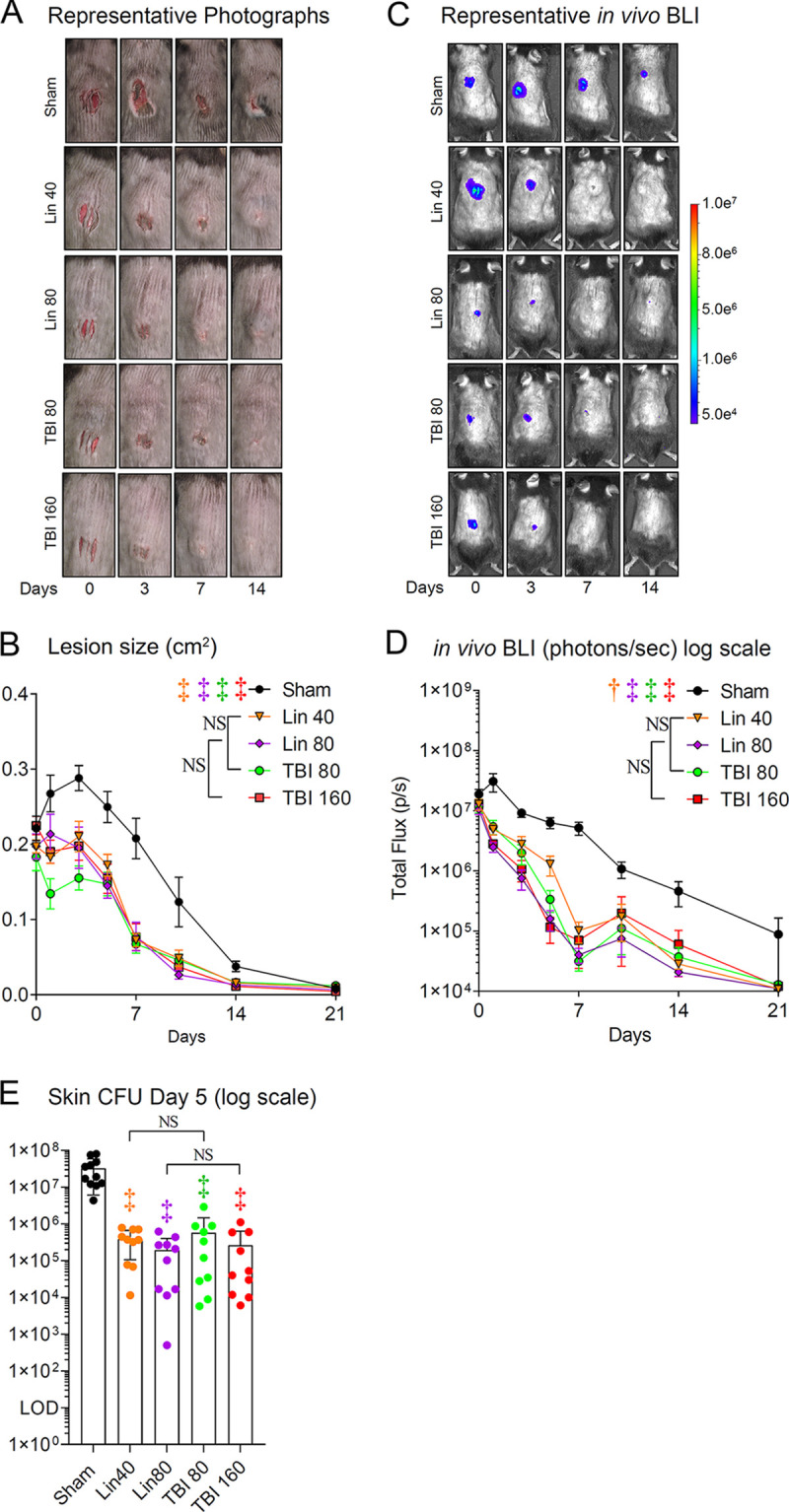
TBI-223 (TBI) versus linezolid (Lin) in a nondiabetic mouse MRSA skin infection model. Three 8-mm-long, parallel, full-thickness scalpel wounds on the backs of C57BL/6 mice (*n* = 10 mice/group) were inoculated with 2 × 10^6^ CFUs of SAP231. Antibiotic treatment was started 4 h postinfection and continued every 12 h for 7 days. (A) Representative photographs of the lesions of the dorsal back. (B) Total lesion size (mean ± SEM). (C) Representative *in vivo* BLI signals on a color scale overlaid on top of a grayscale image of the mice. (D) *In vivo* bacterial burdens as measured by *in vivo* BLI (total flux [mean ± SEM] on a logarithmic scale). (E) CFUs (mean ± SEM on a logarithmic scale) isolated from 8-mm punch biopsy specimens of lesional skin taken on day 5. Combined data from two independent experiments are shown for panels B, D, and E. The limit of detection (LOD) was 10 CFUs per skin biopsy specimen. †, *P < *0.01; ‡, *P < *0.001, antibiotic versus sham treatment groups (symbols) or the indicated groups (brackets) by two-way ANOVA (B and D) or one-way ANOVA (E), both adjusted for multiple comparisons. NS, nonsignificant.

The efficacies of TBI-223 and linezolid were compared in a MRSA skin wound infection model (27). Full-thickness scalpel cuts were performed along the dorsal skin of mice, which were inoculated with 2 × 10^6^ CFU of SAP231 and treated with linezolid or TBI-223 or sham treated for 7 days; the experiment was arbitrarily terminated on day 14. To noninvasively monitor bacterial burden, *in vivo* bioluminescent imaging (BLI) was used to detect signals emitted from MRSA strain SAP231, which highly correlate (*R*^2^ = 0.9996) with CFU counts, from the skin wounds at various time points after infection (28). All experimental groups of antibiotic-treated mice (linezolid [40 and 80 mg/kg] and TBI-223 [80 and 160 mg/kg]) had significantly reduced lesion sizes (Fig. 2A and B) and BLI signals (Fig. 2C and D), compared with sham-treated mice (*P < *0.001 for all comparisons except *P < *0.01 for BLI signals for linezolid [40 mg/kg]-treated versus sham-treated mice). To confirm the *in vivo* BLI data, skin tissue was harvested and CFUs from day 5 skin were enumerated. There were statistically significant decreases in CFUs in all experimental groups of antibiotic-treated mice (linezolid [40 and 80 mg/kg] and TBI-223 [80 and 160 mg/kg]), compared with sham-treated mice (*P < *0.001) (Fig. 2E). However, there were no statistically significant differences in comparisons of 80 versus 160 mg/kg and 40 versus 80 mg/kg linezolid versus TBI-223, respectively.

### Efficacies of TBI-223 and linezolid in a diabetic mouse model of skin wound infection.

**FIG 3:**
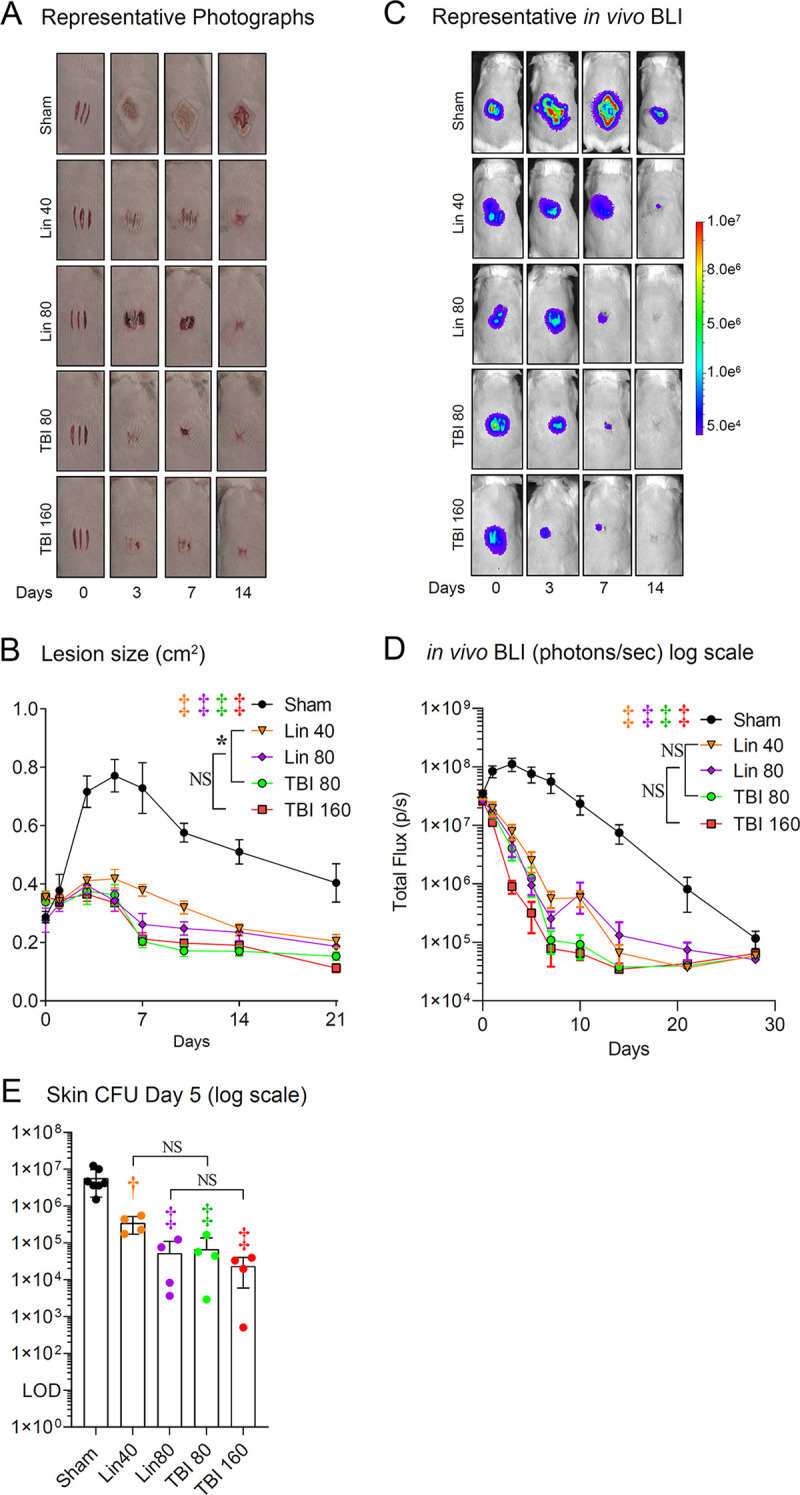
TBI-223 (TBI) versus linezolid (Lin) in a diabetic mouse MRSA skin infection model. Three 8-mm-long, parallel, full-thickness scalpel wounds on the backs of diabetic TallyHo/JngJ mice (*n* = 10 mice/group) were inoculated with 2 × 10^6^ CFUs of SAP231. Antibiotic treatment was started 4 h post-infection and continued every 12 h for 7 days. (A) Representative photographs of the lesions of the dorsal back. (B) Total lesion size (mean ± SEM). (C) Representative *in vivo* BLI signals on a color scale overlaid on top of a grayscale image of the mice. (D) *In vivo* bacterial burdens as measured by *in vivo* BLI (total flux [mean ± SEM] on a logarithmic scale). (E) CFUs (mean ± SEM on a logarithmic scale) isolated from 8-mm punch biopsy specimens of lesional skin taken on day 5. Combined data from two independent experiments are shown for panels B and D. The limit of detection (LOD) was 10 CFUs per skin biopsy specimen. *, *P < *0.05; †, *P < *0.01; ‡, *P < *0.001, antibiotic versus sham treatment groups (symbols) or the indicated groups (brackets) by two-way ANOVA (B and D) or one-way ANOVA (E), both adjusted for multiple comparisons. NS, nonsignificant.

We evaluated the efficacy of TBI-223 and linezolid in the same skin wound infection model using TallyHo/JngJ mice, which develop a disease similar to type 2 diabetes mellitus in humans (29). As we described previously (30), sham-treated diabetic TallyHo/JngJ mice had delayed wound healing, as measured by lesion size (Fig. 3A and B) and increased BLI signals (Fig. 3C and D), compared to nondiabetic mice (Fig. 2A to D). However, similar to the nondiabetic wild-type C57BL/6 mice in Fig. 2, all experimental groups of antibiotic-treated mice (linezolid [40 and 80 mg/kg] and TBI-223 [80 and 160 mg/kg]) had statistically significant reductions in skin lesion sizes (Fig. 3A and B) and *in vivo* BLI signals (Fig. 3C and D), compared with sham-treated diabetic mice (*P < *0.001). Of note, in the diabetic TallyHo/JngJ mice there was a statistically significant difference in lesion size for the 40 mg/kg dose of linezolid versus the 80 mg/kg dose of TBI-223 (*P < *0.05) (Fig. 3B). This difference was not evident when high doses of linezolid and TBI-223 (80 and 160 mg/kg, respectively) were compared. The significant reductions in bacterial burdens in linezolid- and TBI-223-treated mice, compared to sham-treated mice, were validated by CFU enumeration from infected skin samples harvested on day 5 post-infection (Fig. 3E). There were statistically significant decreases in CFUs in all experimental groups of antibiotic-treated mice (linezolid [40 and 80 mg/kg] and TBI-223 [80 and 160 mg/kg]), compared with sham-treated mice (*P < *0.001 for all comparisons except *P < *0.01 for CFUs for linezolid [40 mg/kg]-treated versus sham-treated mice) (Fig. 3E). There were no statistically significant differences in CFU counts in comparisons of 80 versus 160 mg/kg and 40 versus 80 mg/kg linezolid versus TBI-223, respectively.

### Efficacies of TBI-223 and linezolid in a mouse model of S. aureus OIAI.

**FIG 4:**
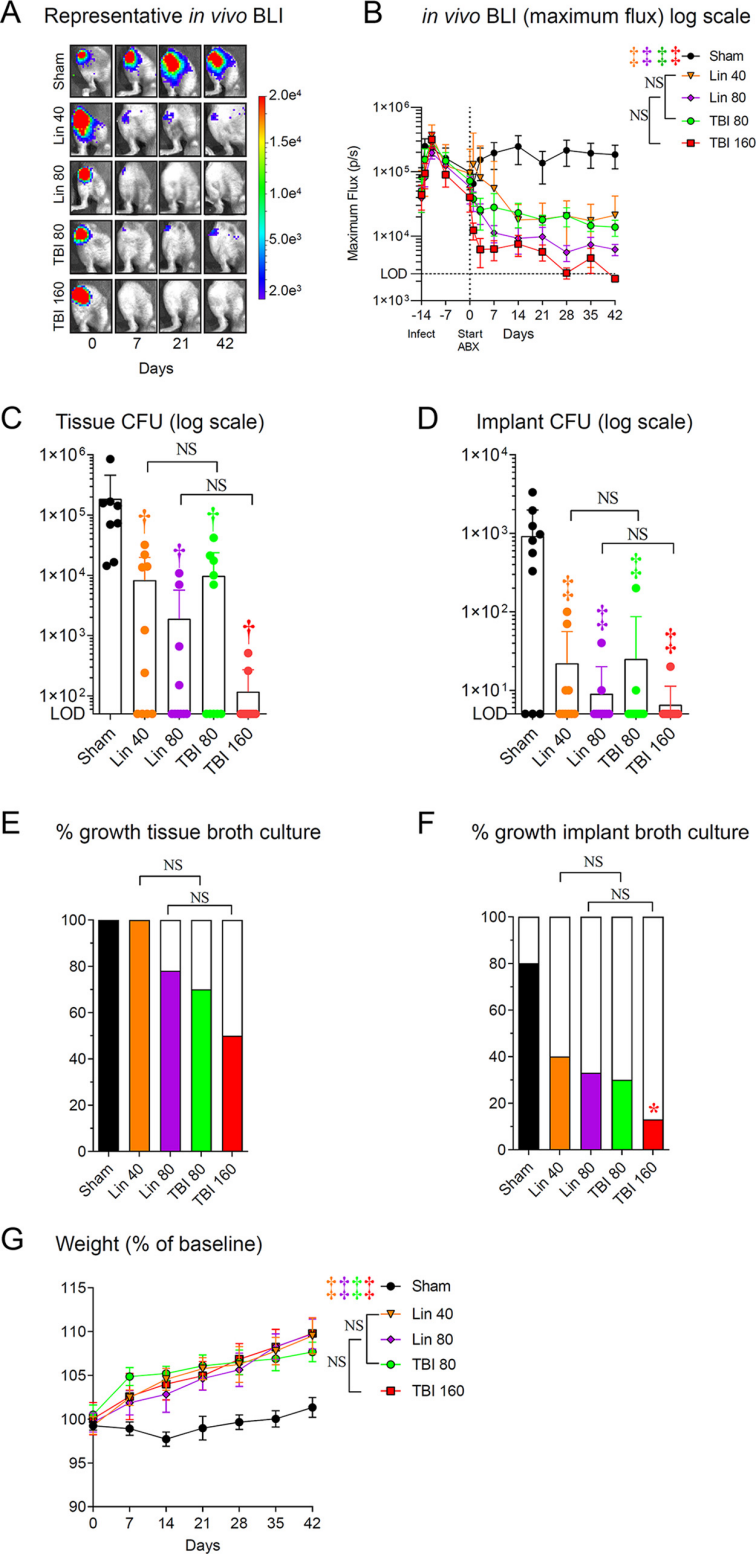
TBI-223 (TBI) versus linezolid (Lin) in a MRSA OIAI mouse model. The OIAI model was established, and antibiotic or sham treatments were initiated 2 weeks after surgery and MRSA inoculation (day 0) and continued for 6 weeks of treatment. Data from a single experiment are presented (*n* = 10 mice/group). (A) Representative *in vivo* BLI images. (B) Maximum flux (mean ± SEM). LOD, limit of detection (2.5 × 10^3^ photons/s/cm^2^/sr). (C and D) CFUs (mean ± SEM on a logarithmic scale) isolated from the peri-implant tissue (C) and implant (D) from euthanized mice after the experiment was ended at 6 weeks. (E and F) Percentages of tissue samples (E) or implants (F) with bacterial growth after tissue homogenates and implants were cultured for an additional 48 h in broth. (G) Body weight versus baseline (mean ± SEM). The limit of detection was 10 CFUs per joint and 1 CFU per implant. *, *P < *0.05; †, *P < *0.01; ‡, *P < *0.001, antibiotic versus sham treatment groups (symbols) or the indicated groups (brackets) by two-way ANOVA (B and G) or one-way ANOVA (C and D) or one-tailed Fisher exact test (E and F), all adjusted for multiple comparisons. NS, nonsignificant.

The efficacies of TBI-223 and linezolid were compared in a mouse model of OIAI, whereby an orthopedic-grade Kirschner wire (K-wire) was surgically placed in the distal femoral intramedullary canal and MRSA (SAP231 [1 × 10^3^ CFU]) was inoculated onto the implant in the knee joint prior to closure, as described previously (31, 32). Linezolid and TBI-223 treatments were initiated 14 days after surgery and MRSA inoculation, and the experiment was arbitrarily ended on day 42. All experimental groups of antibiotic-treated mice (linezolid [40 and 80 mg/kg] and TBI-223 [80 and 160 mg/kg]) had statistically significant reductions in bacterial burdens over the entire duration of the experiment, as measured by *in vivo* BLI, compared with sham-treated mice (*P < *0.001) (Fig. 4A and B). There were no statistically significant differences in comparisons of 80 versus 160 mg/kg and 40 versus 80 mg/kg linezolid versus TBI-223, respectively.

On day 42, infected joint tissue and implants were homogenized and sonicated, respectively, and CFUs were enumerated (Fig. 4C and D). Similar to BLI signals from infected mice (Fig. 4A and B), the infected joint tissues of linezolid (40 and 80 mg/kg)-treated and TBI-223 (80 and 160 mg/kg)-treated mice all had statistically significant reductions in CFUs, compared with those of sham-treated mice (*P < *0.01) (Fig. 4C). In addition, the infected implants of linezolid (40 and 80 mg/kg)-treated and TBI-223 (80 and 160 mg/kg)-treated mice had statistically significant lower CFU counts, compared to sham-treated mice (*P < *0.001) (Fig. 4D). For the CFU counts for both joint tissue and implants, there were no statistically significant differences in comparisons of 80 versus 160 mg/kg and 40 versus 80 mg/kg linezolid versus TBI-223, respectively (Fig. 4A to D). To determine whether linezolid and TBI-223 treatment eradicated the infection, the homogenized joint tissues and sonicated implants from infected mice were cultured in broth for 48 h at 37°C, followed by overnight culture on plates, as a method to evaluate the presence of any S. aureus CFUs, including slow-growing small-colony variants (33). Bacterial growth was present in 100% of the joint tissue samples from sham-treated and linezolid (40 mg/kg)-treated mice. While all of the antibiotic-treated experimental groups had decreased percentages of samples with bacterial growth, only TBI-223 (160 mg/kg)-treated mice had a statistically significant decrease in samples taken from implants, compared with sham-treated mice (*P < *0.05) (Fig. 4E and F). Furthermore, all experimental groups of antibiotic-treated mice (linezolid [40 and 80 mg/kg] and TBI-223 [80 and 160 mg/kg]) had statistically significant increases in body weight, compared with sham-treated mice (*P < *0.001) (Fig. 4G).

**FIG 5:**
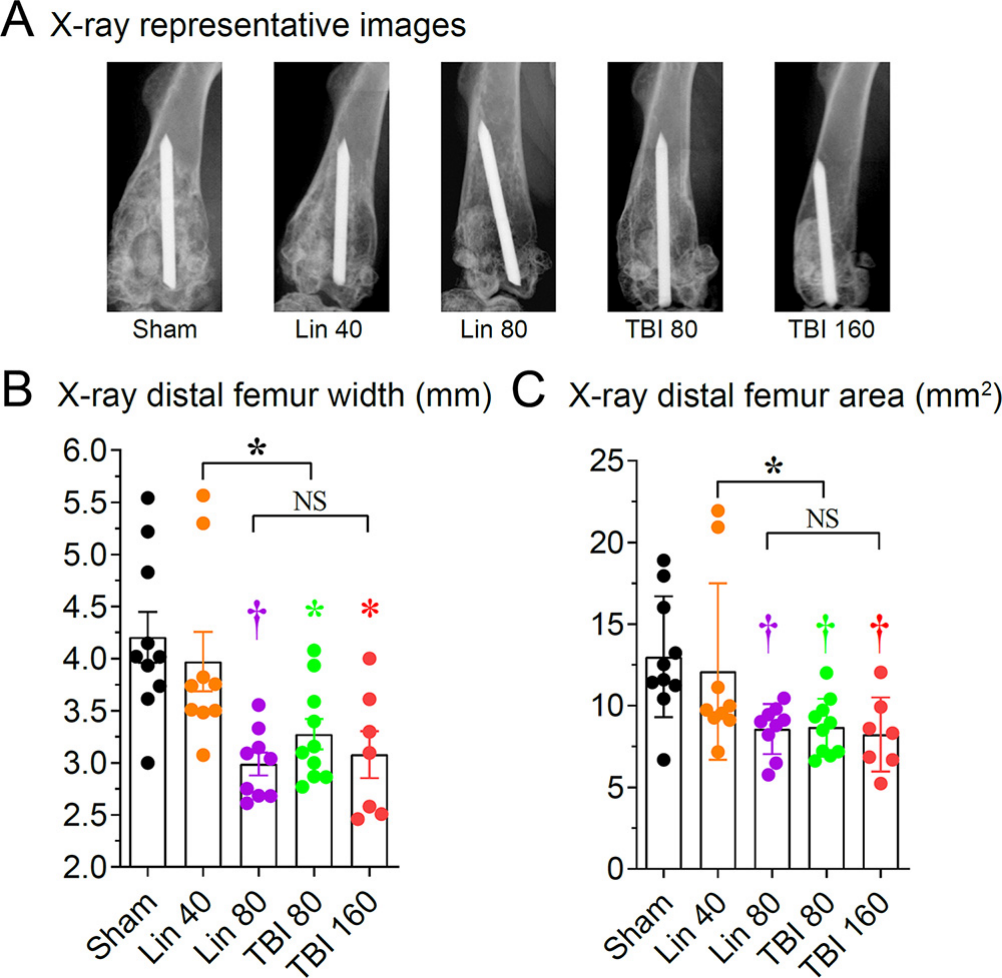
Bone changes with TBI-223 (TBI) versus linezolid (Lin) in a MRSA OIAI mouse model: Radiographs were taken at 6 weeks after antibiotic or sham treatment. Data from a single experiment are presented (*n* = 7 to 10 mice/group). (A) Representative anteroposterior radiographs. (B) Maximum width of the distal 25% of the femur (mean ± SEM). (C) Area of the distal 25% of the femur (mean ± SEM). *, *P < *0.05; †, *P < *0.01; ‡, *P < *0.001, antibiotic versus sham treatment groups (symbols) or the indicated groups (brackets) by one-way ANOVA (B and C), adjusted for multiple comparisons. NS, nonsignificant.

Finally, OIAI causes reactive bone changes associated with periprosthetic osteolysis and cortical expansion of the distal femur in this model (34, 35). Therefore, the impact of the antibiotic treatments on bone morphology on day 42 of the OIAI was examined using high-resolution anteroposterior radiographs of the distal part of the femur (Fig. 5A). Of the antibiotic treatment experimental groups, mice treated with high-dose linezolid (80 mg/kg) and both doses of TBI-223 (80 and 160 mg/kg) had statistically significant reductions in femur width (*P < *0.01 for linezolid [80 mg/kg] and *P < *0.05 for TBI-223 [80 and 160 mg/kg]) and area (*P < *0.01 for all comparisons), compared with sham-treated controls (Fig. 5B and C). Notably, linezolid at 40 mg/kg had femur width and area comparable to those of sham-treated control mice, and there were no statistically significant differences. Moreover, there were statistically significant differences in comparisons of 40 mg/kg linezolid versus 80 mg/kg TBI-223 (*P < *0.05) but not in comparisons of 80 mg/kg linezolid versus 160 mg/kg TBI-223.

### Bone marrow toxicity of the antibiotics.

To evaluate bone marrow toxicity, mice were treated with either linezolid (80 mg/kg) or TBI-223 (160 mg/kg) for 6 weeks and complete blood counts were compared with those of naive untreated mice. We found that linezolid-treated mice had a statistically significant reduction in white blood cell counts, compared to naive and TBI-223-treated mice, whereas there was no significant difference between naive and TBI-223-treated mice (see Fig. S2A). In contrast, there was no difference in polymorphonuclear cell counts among all three groups (see Fig. S2B). Similar to white blood cell counts, linezolid-treated mice had a statistically significant reduction in hemoglobin levels, compared to naive and TBI-223-treated mice, whereas there was no significant difference between naive and TBI-223-treated mice (see Fig. S2C). Finally, we found that linezolid-treated mice had a significant increase in platelets, compared to naive but not TBI-223-treated mice (see Fig. S2D).

## DISCUSSION

Oxazolidinones such as linezolid are an antibiotic class with efficacy against Gram-positive bacteria, including MRSA (2). However, linezolid use is limited by toxicity (especially bone marrow toxicity) when given for prolonged courses (2, 3). Prior studies suggested that TBI-223, a novel oxazolidinone, has reduced toxicity (17, 18). Therefore, we examined the efficacy of TBI-223 in comparison with linezolid in multiple MRSA infection models in mice. We found that TBI-223 had efficacy comparable to that of linezolid in mouse models of bacteremia, nondiabetic and diabetic skin wound infection, and OIAI. These findings provide important new insights into the potential use of TBI-223 against MRSA infections.

First, we found that TBI-223 had significant efficacy against MRSA infections, including in bacteremia, nondiabetic and diabetic skin wound infections, and OIAI. These results are consistent with prior reports that TBI-223 has activity *in vitro* and *in vivo* against other bacteria, such as drug-sensitive and drug-resistant Mycobacterium tuberculosis (17, 18). However, additional studies are needed to determine the broader efficacy of TBI-223 against other antibiotic-resistant Gram-positive bacteria, including vancomycin-resistant enterococci, penicillin- and ceftriaxone-resistant Streptococcus pneumoniae, and coagulase-negative staphylococci, which are frequently resistant to methicillin (2, 3).

Second, TBI-223 had efficacy comparable to that of linezolid in all of the mouse models of bacteremia, nondiabetic and diabetic skin wound models, and OIAI. Since linezolid treatment is effective against MRSA bacteremia in patients (13), our findings support the interpretation that TBI-223 has the potential to be used as a novel antibiotic therapy to treat MRSA bacteremia in humans. Moreover, oxazolidinone (e.g., linezolid) resistance rates among isolates from human infections have remained extremely low (<0.1% of all isolates) (36), indicating that linezolid and other oxazolidinone antibiotics provide excellent coverage against MRSA. S. aureus is the predominant cause of SSTIs (37), and diabetic patients are highly susceptible to S. aureus skin wound infections that lead to complications and delayed wound healing (38). Our data are consistent with prior reports in which linezolid is effective for the treatment of MRSA SSTIs (8, 14), and linezolid penetration into diabetic wounds provides effective concentrations against MRSA infections (11, 39). Therefore, TBI-223 might be an effective antibiotic therapy against MRSA SSTIs in nondiabetic and diabetic patients.

Finally, in accordance with previous studies in rat and dog models (19, 20), bone marrow toxicity was significantly lower in TBI-223-treated mice than in linezolid-treated mice, even at twice the treatment dose. These results suggest that TBI-223 may serve as an additional antibiotic therapy for the treatment of MRSA infections, especially during prolonged antibiotic courses to treat biofilm-associated infections such as OIAIs and infections associated with other implanted medical devices, which often are caused by S. aureus (12).

Our study has several limitations. First, while we tested the MICs of several S. aureus clinical isolates, including linezolid-resistant strains, additional studies would be needed to identify the source of oxazolidinone resistance (e.g., *cfr*, *optrA*, and *poxtA*) in these isolates. Moreover, the *in vivo* efficacy of TBI-223 was tested against only two MRSA strains, and it is not yet known whether these results could be expanded to other Gram-positive bacteria. Second, while we conducted an initial evaluation of bone marrow toxicity in mice, we did not evaluate other toxicities (e.g., liver and kidney injuries and neuropathy associated with MPS), limiting any definitive conclusions related to potential differences in safety and toxicity between TBI-223 and linezolid. However, ongoing clinical studies are evaluating the safety of TBI-223 in humans (including myelosuppression and neuropathy) (21, 40). Third, we evaluated only two dosing regimens to compare TBI-223 to linezolid and not a wider range of dose fractionation, which will be included in future studies. Fourth, although our study incorporated both females (e.g., bacteremia model) and males (e.g., OIAI and skin models), we did not directly evaluate sex-based differences in drug activity in the models; this will be a focus of future work. Moreover, we evaluated TBI-223 in the context of diabetes. However, future studies comparing linezolid and TBI-223 activity should also include a neutropenic mouse model, especially since linezolid is used clinically in neutropenic patients (41). Fifth, we did not evaluate CFU counts in the spleen and whole blood in the bacteremia model, which might have revealed results different from those noted in the kidneys, liver, and heart. Finally, prior studies found that combination therapy, such as the addition of rifampin to linezolid for the treatment of implant-associated MRSA infections, is particularly effective against biofilm-associated S. aureus infections of implants (35, 42). The combination of TBI-223 plus rifampin was not investigated in this study but will be a focus of our future work.

Taken together, the results of this study indicate that TBI-223 has efficacy similar to that of linezolid for the treatment of MRSA bacteremia, nondiabetic and diabetic SSTIs, and OIAIs in the mouse models evaluated. TBI-223 may be considered an additional treatment against MRSA infections and potentially other Gram-positive bacteria in humans, warranting future studies in larger animal models and clinical trials.

## MATERIALS AND METHODS

### Bioluminescent Staphylococcus aureus strain SAP231.

The bioluminescent USA300 S. aureus strain SAP231 (derived from the community-acquired MRSA strain NRS384, which was isolated from an outbreak in the Mississippi prison system) was used in all experiments and was generated as described previously (25). SAP231 possesses a stably integrated modified *luxABCDE* operon from the bacterial insect pathogen Photorhabdus luminescens and has been used previously to study mouse models of OIAI (31, 43). Live and metabolically active SAP231 bacteria constitutively emit a blue-green light, which is maintained in all progeny without selection.

### Bacterial preparation.

SAP231 was streaked on tryptic soy agar (TSA) plates (tryptic soy broth [TSB] plus 1.5% Bacto agar [BD Biosciences]) and grown overnight at 37°C in a bacterial incubator. Two or three colonies were picked and cultured in TSB at 37°C in a shaking incubator (MaxQ HP 420; Thermo Fisher Scientific) at 240 rpm overnight (16 h), followed by a 1:50 subculture at 37°C for 2 h to obtain mid-logarithmic-phase bacteria. The bacteria were pelleted, washed three times, and resuspended in sterile phosphate-buffered saline (PBS) at the indicated concentrations for each model below. The absorbance at 600 nm (*A*_600_) was measured to estimate the number of CFU, which was verified after overnight culture on TSA plates.

### Mice.

C57BL/6 or TallyHo/JngJ mice were used in all experiments (as indicated below) and obtained from the Jackson Laboratory (Bar Harbor, ME). TallyHo/JngJ mice were used as a model of type 2 diabetes and were confirmed to have hyperglycemia (blood glucose levels of >300 mg/dL) before use in experiments, which occurred when the mice were between 8 and 9 weeks of age. There were 10 mice in each group for the bacteremia and OIAI models and 5 mice in each group for the wound infection models. The bacteremia and wound infection models were repeated at least two times to confirm the results. Thus, the total number of mice in the study was 275. Each cage of 5 mice was randomly assigned to a treatment group. Body weight and mouse behavior, posture, and activity level were used to assess drug intolerance or toxicity. There were no significant differences in initial body weights among the groups. All mice were bred and maintained under specific-pathogen-free conditions at an Association for Assessment and Accreditation of Laboratory Animal Care (AAALAC)-accredited animal facility at Johns Hopkins University and were housed according to procedures described in the *Guide for the Care and Use of Laboratory Animals* (44).

### MRSA bacteremia model.

All animal experiments were approved by the Johns Hopkins University Animal Care and Use Committee. The S. aureus bacteremia model was modified from a previously described protocol (26). Briefly, 6-week-old female C57BL/6 mice were anesthetized (inhalation of 2% isoflurane) and inoculated IV with either SAP231 or AR-997 (see Table S3) in a 100-μL volume of PBS using a 29-gauge insulin syringe via the retro-orbital vein. Miller et al. used 1 × 10^7^ CFU to achieve the 30% lethal dose (LD_30_) (26). Here, we used 5 × 10^7^ CFU to achieve the LD_90_ for SAP231 and the LD_50_ for AR-997. Antibiotic treatment (or sham treatment with vehicle solution) was initiated 4 h after inoculation and continued for 7 days, as detailed below.

### MRSA wound infection model.

The S. aureus wound infection model was used as described previously (30). Briefly, 8- to 9-week-old male C57BL/6 mice or TallyHo/JngJ mice were anesthetized via inhalation of isoflurane (2%), the posterior upper backs were shaved, and three parallel 8-mm linear full-thickness scalpel cuts were made with a scalpel with a number 11 blade (30). The wounds were then inoculated with 2 × 10^6^ CFU of SAP231 using a micropipette. Antibiotic treatment (or sham treatment with vehicle solution) was initiated 4 h after inoculation and continued for 7 days, as detailed below. The total lesion size (in square centimeters) was quantified from digital photographs by using the image analysis software program ImageJ (National Institutes of Health [NIH] Research Services Branch) (http://rsbweb.nih.gov/ij) with a millimeter ruler as a reference.

### MRSA OIAI model.

The OIAI surgical procedure was modified from a previously described protocol (31, 45). Briefly, 10- to 12-week-old C57BL/6 male mice were anesthetized via inhalation of isoflurane (2%), a medial parapatellar approach was performed, and the patella was dislocated laterally to access the distal part of the femur. The femoral medullary canal was reamed with a 25-gauge needle, and a surgical-grade titanium K-wire (0.5 in diameter and 9 mm in length; Modern Grinding) was inserted in a retrograde fashion with 1 mm protruding into the knee joint. An inoculum of 1 × 10^3^ CFU of SAP231 in 2 μL of PBS was pipetted onto the exposed K-wire in the knee joint before closure with absorbable sutures. For pain management, sustained-release buprenorphine (2.5 mg/kg) was administered subcutaneously at the time of surgery. After a 2-week incubation period to allow biofilm formation on the K-wire (31), antibiotic treatment was initiated for 6 weeks, as detailed below. Mice were closely monitored and would have been prematurely euthanized if they had developed signs of severe systemic infection (e.g., an inability to obtain food or water, extreme lethargy, hypothermia, an inability to remain upright, respiratory distress, or dehydration). No animal (antibiotic treated or sham treated) developed signs of severe systemic infection, and all animals were euthanized at the completion of the experiment.

### PK analyses.

All PK analyses were performed by BioDuro Inc. (Beijing, China). Briefly, oral administration of 100 mg/kg of either linezolid or TBI-223 to BALB/c mice (*n* = 3 per drug) was followed by blood sampling at 15 and 30 min and 1, 2, 4, 7, and 24 h post-dose. Plasma was isolated and subjected to liquid chromatography-tandem mass spectrometry (LC-MS/MS) using the API 4000 platform (AB Sciex, USA) for quantification of the antibiotic of interest using multiple reaction monitoring (see Tables S1 and S2 in the supplemental material). Calculation of the AUC, *t*_1/2_, and maximum drug concentration (*C*_max_) was performed using Phoenix WinNonLin PK software v6.4 (Certara, USA).

### MIC determinations.

Ten clinical isolates were received from the Centers for Disease Control and Prevention (CDC) collections of tedizolid/linezolid (oxazolidinones)-resistant staphylococci and difficult-to-detect Staphylococcus aureus harboring *mecA*. The MICs against these strains (*n* = 2 repeats/antibiotic) and against SAP231 (*n* = 3 repeats/antibiotic) were determined by broth microdilution assays (46). Briefly, bacteria were grown to log phase in cation-adjusted Mueller-Hinton broth (CAMHB). Linezolid or TBI-223 at 256 μg/mL was serially diluted on a 96-well plate, and bacteria were added (10^4^ CFU/well). A negative control with no bacteria and a positive control with no antibiotics were used to validate the assay. Growth was evaluated after 16 to 20 h of culture at 35°C in a bacterial incubator. MIC was defined as the lowest concentration at which no visible growth occurred. Linezolid MICs for the clinical strains generally matched those reported by the CDC (47).

### TBI-223 and linezolid treatment.

TBI-223 and linezolid (both kindly provided by the TB Alliance [New York, NY]) in powder form were dissolved in water containing 0.5% methylcellulose (sham treatment) by first mashing the powder into a paste, followed by sonication for 5 min. A 200-μL volume of TBI-223 (80 mg/kg or 160 mg/kg), linezolid (40 mg/kg or 80 mg/kg), or sham treatment was given by oral gavage every 12 h, as indicated for each model. Linezolid doses approximated human equivalent doses according to the matching AUC values for humans and mice. Following a dose of 100 mg/kg, we determined the linezolid AUC in mice to be 130.7 μg · h/mL. Assuming dose proportionality, the corresponding AUC for a dose of 80 mg/kg given twice daily to mice is 209.12 μg · h/mL, which is equivalent to the reported AUC of 228 μg · h/mL for a human clinical dose of 600 mg administered twice daily (10, 22–24).

### *In vivo* BLI and analysis.

To noninvasively and longitudinally monitor bacterial burdens, *in vivo* BLI using the IVIS Lumina Series III imaging system (PerkinElmer) was performed on anesthetized mice (2% isoflurane) at the indicated time points. Bioluminescent signals were localized on a grayscale image of the mice and quantified within an oval region of interest (0.5 by 0.75 cm), using either total flux (photons per second) or maximum flux (photons per second per square centimeter per steradian) for the skin and OIAI models, respectively (level of detection, 2.5 × 10^3^ photons/s/cm^2^/sr).

### CFU enumeration.

To measure bacterial burdens in all of the mouse models, enumeration of CFU was performed with overnight plate cultures of homogenized tissue (Pro200 Series homogenizer; Pro Scientific, Oxford, CT). For the bacteremia model, whole organs, including heart, liver, and both kidneys, were harvested on day 3 as described previously (26). Bilateral kidneys were processed together. For the nondiabetic skin infection model, 8-mm punch biopsy specimens of lesional skin were taken on day 5. For the OIAI model, mice were euthanized 1 day after the 6-week antibiotic course. To avoid disturbing the biofilm at the distal end of the implant, the surrounding bone was gently crushed circumferentially with a needle driver and the implant was carefully extracted. Bacteria were isolated from the bone and joint tissue by homogenizing tissue from the midpart of the femur through the proximal part of the tibia. Bacteria adherent to the K-wire implants were isolated by sonication in 0.3% Tween solution (Sigma-Aldrich) for 10 min, followed by vortex-mixing for 2 min. The CFU were counted after overnight culture with serial dilutions on plates. To further identify any bacteria remaining in the bone or joint tissue or the implants, homogenates and sonicates were cultured for an additional 48 h at 37°C in TSB at 240 rpm, and the presence or absence of bacterial growth was determined after overnight culture on plates.

### Complete blood count assay.

Mice were treated with either linezolid (80 mg/kg/dose) or TBI-223 (160 mg/kg/dose) every 12 h for 6 weeks. Thereafter, mice were sacrificed, and blood was collected in Microtainer tubes containing EDTA. Untreated mice served as naive controls. An automated complete blood count with differential was performed (Department of Pathology, Johns Hopkins Hospital).

### Radiographic imaging.

After the 6-week antibiotic course and immediately before tissues were harvested for CFU counts, anteroposterior high-resolution radiographs were obtained using the Faxitron MX-20 X-ray system (Faxitron Bioptics). Radiographs were analyzed with ImageJ (NIH Research Services Branch) (https://imagej.nih.gov/ij) by an observer blinded to the treatment groups. The maximal femoral width (proximal to the fabella) was measured perpendicular to the anatomical axis of the distal part of the femur. The area of the distal 25% of the femur (from the midpoint of a line extending from the intercondylar notch to its intersection with a perpendicular line that bisected the third trochanter) was also calculated.

### Statistics.

GraphPad Prism 9 (GraphPad Software) was used to compare data among groups. Survival rates were compared by log rank (Mantel-Cox) test. *In vivo* BLI signals were compared using two-way analysis of variance (ANOVA). CFU counts, blood indices, and radiographic findings were compared using one-way ANOVA. R software v4.0.2 (R Core Team) was used to compare the presence or absence of bacterial growth in broth. Comparisons between treatment groups utilized a two-tailed Fisher exact test, and comparisons between treatment groups and the sham treatment utilized a one-tailed Fisher exact test. The one-tailed Fisher exact test was chosen assuming reduced growth in treatment groups, compared to sham treatment. *P* values of <0.05 were considered statistically significant. Analyses were adjusted for multiple comparisons to preserve the false-discovery rate (48).
