# Supplementary material for: The Novel Oxazolidinone TBI-223 Is Effective in Three Preclinical Mouse Models of Methicillin-Resistant Staphylococcus aureus Infection
Source: Microbiol Spectr. 2022 Sep 15;10(5):e02451-21. doi: 10.1128/spectrum.02451-21 (PMC9603142; doi:10.1128/spectrum.02451-21)
Supplement: Supplemental file 1 — Fig. S1 and S2; Tables S1 to S3. Download spectrum.02451-21-s0001.pdf, PDF file, 0.4 MB [file spectrum.02451-21-s0001.pdf]

The novel oxazolidinone TBI-223 is effective in three preclinical mouse models of methicillin-resistant *Staphylococcus aureus* infection

Supplementary material

Running title: TBI-223 is effective against MRSA infections in mice

Authors: Oren Gordon[1], Dustin A. Dikeman[2], Roger V. Ortines[2], Yu Wang[2], Christine Youn[2], Mohammed Mumtaz[2], Nicholas Orlando[2], Jeffrey Zhang[2], Aman Patel[2], Ethan Gough[3], Amit Kaushik[4], Eric L. Nuermberger[4], Anna M. Upton[5], Nader Fotouhi[6], Lloyd S. Miller[2,7], Nathan K. Archer[2,*]

Affiliations:
[1]Division of Infectious Diseases, Department of Pediatrics, Johns Hopkins University School of Medicine, Baltimore, MD 21287, USA.

[2]Department of Dermatology, Johns Hopkins University School of Medicine, Baltimore, MD 21231, USA.

[3]*Department of International Health, Johns Hopkins Bloomberg School of Public Health, Baltimore, MD, 21205, USA.*

[4]Center for Tuberculosis Research, Department of Medicine, Johns Hopkins University School of Medicine, and Department of International Health, Johns Hopkins Bloomberg School of Public Health, Baltimore, MD 21231-1002.

[5]Evotec (US) Inc., 303b College Rd E, Princeton, NJ 08540.

[6]TB Alliance, New York, NY.

[7]Immunology, Janssen Research and Development, Spring House, PA 19477, USA

*Corresponding Author:

Nathan K. Archer, Ph.D.

Johns Hopkins Department of Dermatology

Cancer Research Building II, Suite 2M04

1550 Orleans Street

Baltimore, MD 21231

Phone: (410) 614-3490

Fax: (410) 955-8645

Email: narcher2@jhmi.edu

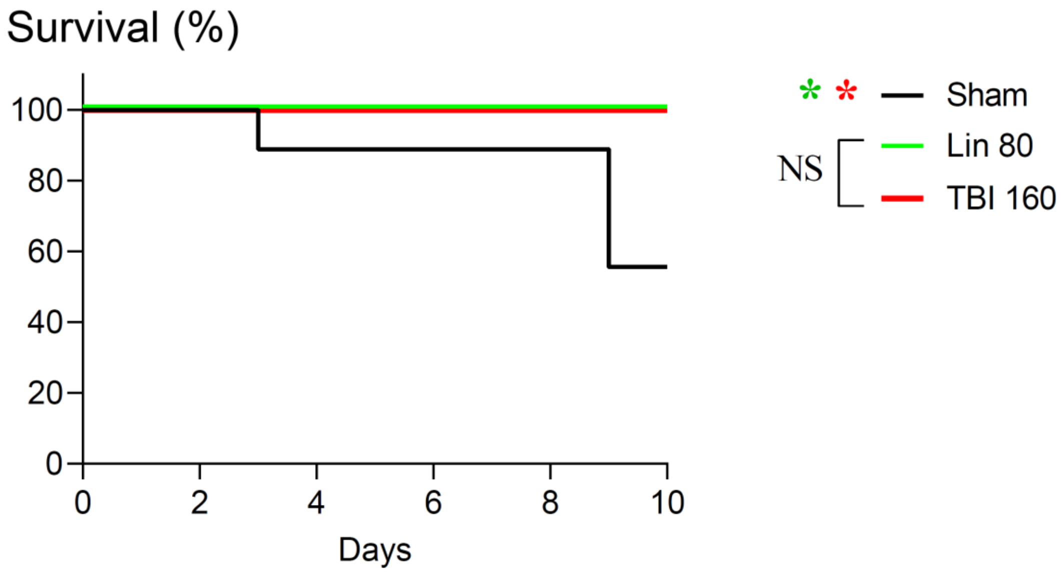

Supplementary Figure 1. TBI-223 versus linezolid in a MRSA bacteremia mouse model. Mice were inoculated with 5×10^7 CFU of clinical isolate AR-997 (see Table 3) i.v. via the retro-orbital vein. Antibiotic treatment was started 4 hours post-infection and continued every 12 hours up to 7 days. Treatment groups included linezolid (Lin) 80 mg/kg/dose, TBI-223 (TBI) 160 mg/kg/dose and sham treatment (vehicle). Kaplan Meier graph of percent survival (results of one experiment; n=7-9 mice/group). *P<0.05, between antibiotic versus sham-treatment groups (color * symbols) or indicated groups (brackets) by the log-rank (Mantel-Cox) test adjusted for multiple comparisons to preserve the desired false discovery rate. NS=non-significant.

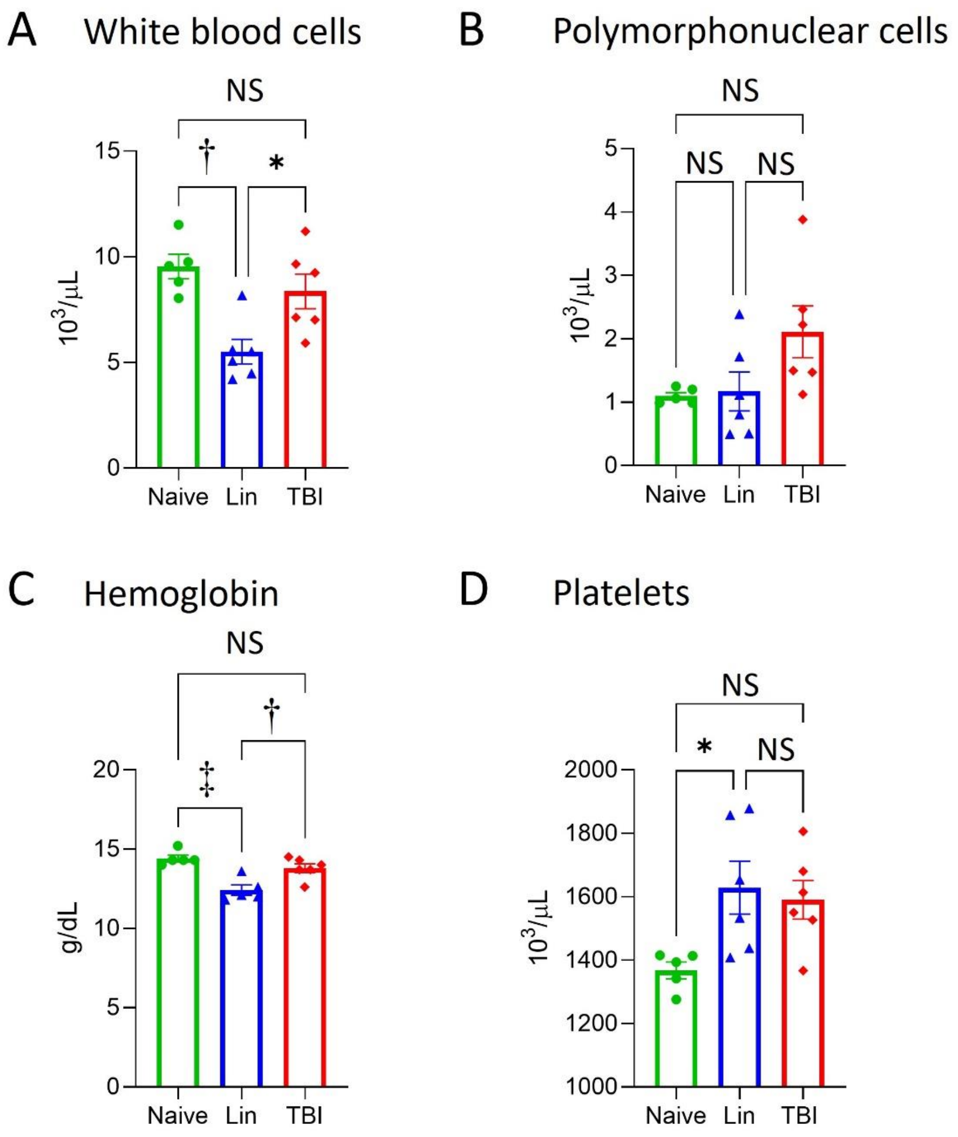

Supplementary Figure 2. Bone marrow suppression due to linezolid versus TBI-223. Mice were treated with either linezolid 80 mg/kg/dose (Lin) or TBI-223 160 mg/kg/dose (TBI) every 12 hours for 6 weeks. Untreated mice served as controls (naïve). Complete blood count was taken at the end of treatment and mean (± SEM) white blood cells (A), polymorphonuclear cells (B), hemoglobin (C) and platelets (D) are shown (results of one experiment; n=5-6 mice/group). *P<0.05 and †P<0.01, ‡P<0.001 between the groups as indicated by one-way ANOVA corrected for multiple comparisons. NS=non-significant.

Supplementary Table 1. Pharmacokinetic analysis of linezolid in mice.

**Matrix**	**Plasma**	**Plasma retest**
Standard Range	10-10000 ng/mL	1-1000 ng/mL
Regression	Linear	Linear
Weighting	1 / (x * x)	1 / (x * x)
LLOQ	10 ng/mL	1 ng/mL
Internal Standard	Terfenadine (50 ng/mL) in Acetonitrile	Terfenadine (50 ng/mL) in Acetonitrile
**%CV for internal standard (IS) peak area**	**Initial assay**	**Re-assay**
Average of IS peak area	1.86E+05	1.68E+05
SD	9.31E+03	7.78E+03
**%CV (< ± 30%)**	**5.0**	**4.6**

CV – Coefficient of variation; LLOQ – Lower limit of quantification; SD – Standard deviation.

Supplementary Table 2. Pharmacokinetic analysis of TBI-223 in mice.

**Matrix**	**Plasma**	**Plasma retest**
Standard Range	10-10000 ng/mL	1-2000 ng/mL
Regression	Linear	Linear
Weighting	1 / (x * x)	1 / (x * x)
LLOQ	10 ng/mL	1 ng/mL
Internal Standard	Terfenadine (50 ng/mL) in Acetonitrile	Terfenadine (50 ng/mL) in Acetonitrile
**%CV for internal standard (IS) peak area**	**Initial assay**	**Re-assay**
Average of IS peak area	2.07E+05	7.09E+04
SD	1.10E+04	2.42E+03
**%CV (< ± 30%)**	**5.3**	**3.4**

CV – Coefficient of variation; LLOQ – Lower limit of quantification; SD – Standard deviation.

Supplementary Table 3. Minimal inhibitory concentrations (MIC) for linezolid and TBI-223 against resistant and susceptible *Staphylococcus aureus* clinical isolates as determined by the broth microdilution assay.

Isolate (AR-Bank#)	*mecA*+	Linezolid MIC (µg/ml)	Linezolid interpretation*	TBI-223 MIC (µg/ml)
702	Yes	8	R	16
704	Yes	16	R	32
709	Yes	16	R	64
710	Yes	16	R	64
716	No	>64	R	>64
978	Yes	2	S	8
979	Yes	1	S	4
997	Yes	1	S	4
998	Yes	1	S	4
1005	Yes	1	S	4
SAP231	Yes	1	S	4

*Based on the Clinical and Laboratory Standards Institute (CLSI)
